# Macrophage Migration Inhibitory Factor Polymorphism Is Associated with Susceptibility to Inflammatory Coronary Heart Disease

**DOI:** 10.1155/2015/315174

**Published:** 2015-03-04

**Authors:** Kangting Ji, Xiaoyan Wang, Ji Li, Qin Lu, Guoqiang Wang, Yangjing Xue, Suqin Zhang, Lu Qian, Wenwu Wu, Yongjin Zhu, Luping Wang, Lianming Liao, Jifei Tang

**Affiliations:** ^1^Department of Cardiology, The Second Affiliated Hospital, Wenzhou Medical University, Wenzhou 325000, China; ^2^Department of Pharmacology and Toxicology, School of Medicine and Biomedical Sciences, University at Buffalo, The State University of New York, Buffalo, NY 14214, USA; ^3^Academy of Integrative Medicine, Fujian University of Traditional Chinese Medicine, Huatuo Road, No. 1, Fuzhou 350112, China

## Abstract

*Background*. Macrophage migration inhibitory factor (MIF) is a proinflammatory cytokine. This study explored the association of 173G/C polymorphism of the MIF gene with coronary heart disease (CHD). *Methods*. Sequencing was carried out after polymerase chain reaction with DNA specimens from 186 volunteers without CHD and 70 patients with CHD. Plasma MIF levels on admission were measured by ELISA. Patients were classified into either stable angina pectoris (SAP) or unstable angina pectoris (UAP). Genotype distribution between cases and controls and the association of patients' genotypes with MIF level and plaque stability were statistically evaluated (ethical approval number: 2012-01). *Results*. The frequency of the C genotype was higher in CHD patients than in the control (*P* = 0.014). The frequency of the 173^*^CC genotype was higher in CHD patients than in the control (*P* = 0.005). The plasma MIF level was higher in MIF173^*^C carriers than in MIF173^*^G carriers (*P* = 0.033). CHD patients had higher plasma MIF levels than the control (*P* = 0.000). Patients with UAP had higher plasma MIF levels than patients with SAP (*P* = 0.014). *Conclusions*. These data suggest that MIF −173G/C polymorphism may be related to the development of CHD in a Chinese population. Plasma MIF level is a predictor of plaque stability. This trial is registered with NCT01750502 .

## 1. Introduction

Despite improvements in medical treatments and subsequent survival rates, coronary heart disease (CHD) has become the leading cause of death worldwide, especially in the developed countries [1]. Pathologically CHD is characterized by the growth of atherosclerotic plaques in the vascular wall that results in vascular stenosis or plaque disruption with acute thrombotic occlusion [2]. Various risk factors have been identified for CHD, such as smoking, hypercholesterolemia, hypertension, and diabetes [3]. With the development of society, psychosocial risk factors also contribute to the development of CHD [4]. Recent evidence demonstrates that CHD is an inflammatory process with chronic inflammation of vessel wall infiltrated by circulating immune cells, such as monocytes and macrophages [5]. Sustained inflammation of vessel wall leads to chronic alterations of inflammatory mediators. Interaction between endothelial cells and circulating immune cells also contributes to plaque instability and subsequent atherothrombotic events [6–8]. Indeed, studies have demonstrated an association between markers of inflammation and future coronary risk in both healthy individuals and those with CHD.

Macrophage migration inhibitory factor (MIF) is a homotrimer with a molecular weight of 37.5 kDa. MIF binds to cells by interaction with the extracellular domain of CD74 and initiates ERK-1/2 activation [9]. MIF is expressed in several cell types, including monocytes, macrophages, vascular smooth muscle cells (SMCs), and cardiomyocytes [10–12]. MIF is considered to play a critical role in the development of atherosclerosis [13]. In hyperlipidemic rabbits, markedly upregulated MIF expression during atherogenesis was observed [14]. Throughout lesion formation and progression, vascular endothelial cells displayed an increased MIF expression compared with normal arteries. Also, medial SMCs strongly expressed MIF in early lesions [14]. Similarly, MIF was highly expressed in monocytes adhering to the endothelial cells and in macrophages accumulating in early fatty streaks [14]. Treatment with MIF-neutralizing antibody could decrease accumulation of intimal macrophages and T lymphocytes in Apoe^−/−^ mice [15, 16] and even induced a regression of established atherosclerotic lesions [16]. In accordance with these observations, complete inhibition of MIF activity via a genetic deletion of MIF in low-density lipoprotein receptor-deficient (Ldlr^−/−^) mice resulted in reduced aortic intimal thickening and lipid deposition even when the mice were fed a high-fat diet [17]. In human, MIF was shown to be expressed in different stages of human atherosclerosis [11, 18], markedly upregulated in vulnerable atheromatous plaques, and was associated with the weakening of the fibrous cap [19].

The* MIF* gene maps to chromosome 22q11.2 in human. There are four polymorphisms that have been mainly reported in the human* MIF* gene [20, 21], including three SNPs at positions −173 (rs755622), +254 (rs2096525), and +656 (rs2070766) and a 794CATT5-8 microsatellite polymorphism. Loci rs2096525 and rs2070766 are located in introns, whereas rs755622 and −794CATT5-8 are located in the promoter region of MIF. Polymorphism in the −173C allele has been associated with higher transcription activity of* MIF* gene and increased production of MIF protein [21]. However the CATT5 allele has the lowest level of basal and stimulated MIF promoter activity* in vitro* compared with other alleles [22]. The functional importance of MIF in immune-mediated inflammatory diseases prompted us to evaluate the association of MIF −173G/C polymorphism with the development of CHD.

## 2. Materials and Methods

### 2.1. Subjects

A total of 70 unrelated CHD patients were enrolled in the study, including 44 males and 26 females, in the Second Hospital of Wenzhou Medical College, China. CHD was confirmed by coronary angiography (CAG). Healthy volunteers without CHD (*n* = 186, 84 males and 102 females) served as controls. They were also confirmed by CAG. Participants were all of Han origin living in Wenzhou, a southeastern coastal city of China. None of the patients and volunteers had cancer, inflammatory or autoimmune diseases, diabetes, and liver and kidney dysfunction.

Braunwald classification method was used to further divide the patients into two categories: stable angina pectoris (SAP) and unstable angina pectoris (UAP). Diagnosis criteria of SAP include discomfort behind the sternum that is usually precipitated by stress or exertion and relieved rapidly by rest or nitrates. Diagnosis criteria of UAP include (1) new onset (<2 months) angina that is severe and frequent (≥3 times/day); (2) accelerated angina, that is, angina that is distinctly more frequent, severe, prolonged, or precipitated by less exertion than previously; (3) angina at rest.

### 2.2. Blood Sample and Genomic DNA Extraction

A blood sample of 5 mL was collected in a tube containing ethylene diamine tetraacetic acid (EDTA) from the radial artery. After centrifugation, plasma was collected and stored at −80°C for use. Genomic DNA was extracted from cells by a DNA extraction kit (Tiangen Company, Beijing, China). The isolated DNA was stored at −80°C for use.

### 2.3. MIF 173G/C Genotyping

Polymorphism was genotyped by sequencing of polymerase chain reaction (PCR) product as reported previously [8, 9]. Primers were synthesized by Shanghai GeneCore BioTechnologies Co., Ltd. The forward primer was 5′-ACT AAG AAA GAC CCG AGG C-3′ and the reverse primer was 5′-GGG GCA CGT TGG TGT TTAC-3′. These primers were designed to amplify a 366 bp segment of the MIF promoter region. PCR was carried out in a volume of 25 ul. The reaction conditions of PCR were as follows: initial denaturation at 95°C for 5 min, followed by 35 cycles at 95°C for 30 s, 60°C for 30 s, and 72°C for 1 min, with final extension at 72°C for 10 min. PCR products were confirmed by agarose gel electrophoresis and then sent to Shanghai Hybio BioTechnology Co., Ltd. for sequencing.

### 2.4. ELISA

The plasma concentrations of MIF were measured using an enzyme linked immunosorbent assay (ELISA) kit (R&D, USA).

### 2.5. Statistical Analyses

MIF genotype and allele frequencies were analyzed using SPSS17.0 statistical software. The allele and genotype distributions were detected by Hardy-Weinberg equilibrium (*P* > 0.05). The genotype and allele frequencies for CHD patients and control group individuals were analyzed using *χ* ^2^-test or Fisher's exact test. Odds ratio (OR) with 95% confidence interval (CI) was calculated. *P* ≤ 0.05 was considered statistically significant. Plasma MIF concentrations were expressed as means ± SD. For comparisons between two groups, we determined the significance of differences between means by *t*-tests. Comparisons between multiple groups were performed by ANOVA.

## 3. Results

### 3.1. Frequencies of MIF −173G/C Alleles and Genotypes of CHD Patients and Controls

There were three kinds of genotypes in the two groups: CC, CG, and GG. There were two kinds of alleles in the two groups: G and C. With regard to the −173 polymorphisms, genotype distributions in CHD patients and control group are shown in Table 1. Both CHD patients and controls were in Hardy-Weinberg equilibrium with MIF −173G/C genotypes' distribution (*P* > 0.05). Comparison of the gene frequency distribution showed the frequencies of three genotypes were significantly different between the two groups (*P* = 0.005). The frequencies of the two alleles were also significantly different (*P* = 0.014, OR = 1.81). There was significant difference in the distribution of genotypes CC and CG in CHD patients and controls (*P* < 0.05/4, OR = 5.238) (Table 2). Therefore, CC genotype was a risk factor for CHD compared with CG genotype. Similarly, there was significant difference in the distribution of genotypes CC and GG in CHD patients and the control group (*P* < 0.05/4, OR = 4.928). The results suggested that CC genotype was a risk factor for CHD compared with GG genotype. No significant difference was found for both GG and CG frequencies between CHD patients and controls. Thus MIF173C allele may increase susceptibility to CHD.

### 3.2. The Plasma Concentration of MIF

The plasma MIF concentration of carriers of C allele was 59.12 ± 10.38 ug/L, significantly higher than that of G allele carriers (56.11 ± 9.77 ug/L, *P* = 0.033).

CHD patients were further divided into the stable angina pectoris subgroup (*n* = 10) and the unstable angina pectoris subgroup (*n* = 52) by Braunwald classification method and clinical manifestations. Age, gender, cigarette smoking, drinking, hypertension, HDL-C, LDL-C, TC, and TG were comparable between control group and each CHD subgroup (Table 3). The plasma MIF levels of stable angina pectoris (SAP) subgroup, unstable angina pectoris (UAP) subgroup, and control group were 61.52 ± 4.3 ug/L, 66.79 ± 6.29 ug/L, and 48.08 ± 6.48 ug/L, respectively (Table 3). The plasma MIF concentrations were significantly different among the three groups (*P* = 0.000). In the CHD group, the plasma MIF concentration was higher in the UAP group than in the SAP group (*P* < 0.05).

### 3.3. Receiver Operator Characteristic Curve

**Figure 1:**
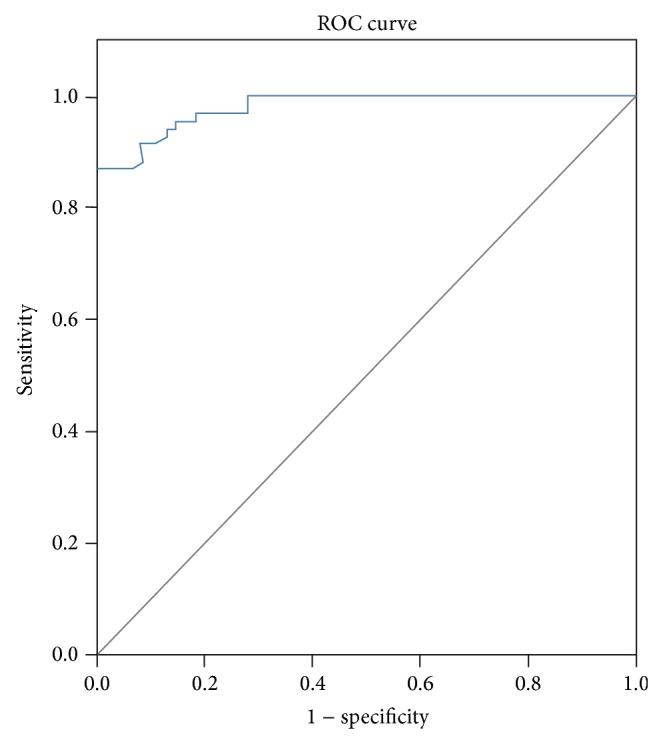


**Figure 2:**
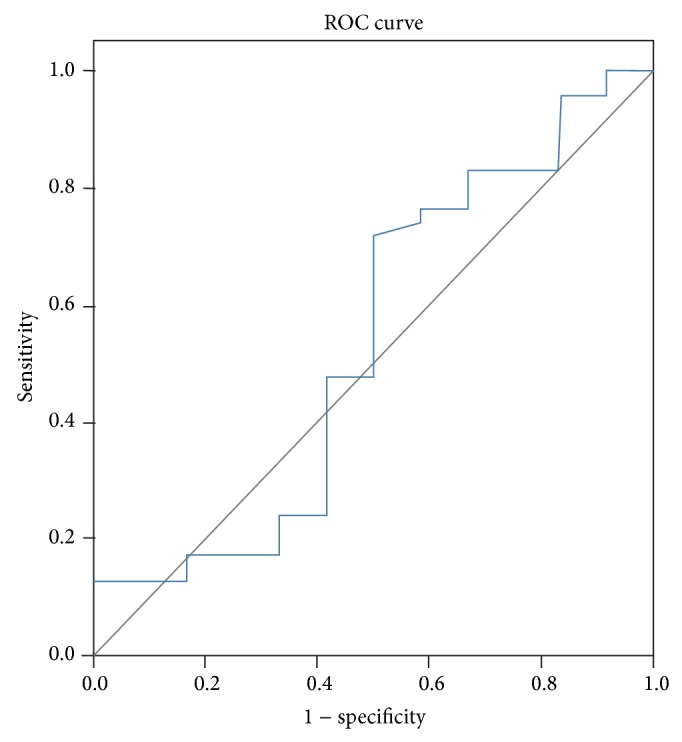


Finally, we performed receiver operator characteristic curve (ROC). The area under the ROC curve was 0.980 (Figure 1), suggesting that the diagnostic value of MIF for coronary heart disease is high. For ROC curve of SAP and UAP plasma concentration of MIF, the area under the ROC curve was 0.535, suggesting that the diagnostic value of MIF for SAP was low (Figure 2).

## 4. Discussion

It has been well known for decades that there are a variety of risk factors contributing to CHD, such as smoking tobacco or disorder in lipid metabolism [23–25]. In addition, it is well documented that there is strong relationship between many genetic variants and CHD as CHD shows strong familial aggregation in some patients [26–30]. However, only a small percentage of CHD patients have recognized monogenetic disease due to dysfunctional mutations [30]. Therefore, various genetic risk factors remain unexplained. Thus further genetic studies are needed to elucidate the pathogenesis of CHD.

In the present study we demonstrate a close association between the polymorphism of MIF on the −173 position and CHD. Polymorphism of MIF on the −173 position has been reported in several diseases, including juvenile idiopathic arthritis (JIA) [31], multiple sclerosis [32], systemic lupus erythematosus (SLE) [33], and ulcerative colitis [34]. The G-to-C transition at −173 is the most likely functional polymorphism identified so far, especially since the presence of the mutant C allele creates an AP-4 transcription factor binding site [35]. On the contrary, intronic polymorphisms of MIF did not result in transcripts of different length for all the tissues studied thus far [36]. Thus the present study focused on the −173 position.

We found that increased risk of susceptibility to CHD in carriers of the MIF −173 ^*^C allele was accompanied by increased plasma MIF concentration. When MIF protein levels were determined in the plasma of CHD and control individuals, those with a MIF −173 ^*^C allele were found to have significantly higher levels of MIF, suggesting that individuals carrying the MIF −173 ^*^C allele produce higher amounts of MIF protein. This is in accordance with previous studies suggesting that healthy individuals with MIF −173 ^*^C allele have significantly higher MIF concentration compared with individuals with the MIF −173 ^*^GG genotype [35].

MIF may have a critical role in the pathogenesis of CHD, which is mainly caused by atherosclerosis. It has become increasingly clear that inflammation and immunity are tightly associated with atherogenesis. Monocytes/macrophages, T cells, neutrophils, and dendritic cells call contribute to the development and progression of atherosclerosis [13]. MIF may activate macrophage and induce hemorrhagic microvessels in atherosclerosis [37]. Markedly upregulated MIF expression during atherogenesis was observed in the vascular endothelial cells, monocytes adhering to the endothelial cells, macrophages accumulating in early fatty streaks, and atheromatous plaques [11, 14, 16, 18, 19]. Because MIF inhibition has been shown to induce the stabilization and even regression of atherosclerotic plaques, MIF represents a potential drug target for the treatment of atherosclerosis [15, 16].

Increased MIF protein expression has also been observed in a plethora of diverse immune-related diseases. In experimental colitis, MIF-deficient mice failed to develop disease, but reconstitution of MIF-deficient mice with wild-type innate immune cells restored colitis. In addition, established colitis could be treated with anti-MIF immunoglobulins. Thus continuous MIF production by the innate immune system is critical for murine colitis development [38]. Furthermore, increased MIF concentrations were found in patients with Crohn's disease [36]. The MIF levels in patients with psoriasis were also found to be significantly higher than those in controls. Both spontaneous and concanavalin-A induced MIF production of peripheral blood mononuclear cells from psoriatic patients were significantly higher compared to healthy controls [39]. Rheumatoid arthritis is also characterized by an increased activity of MIF. MIF is abundantly expressed in the plasma and synovial tissue of rheumatoid arthritis patients where it correlates with disease activity. In multiple rat and mouse models of rheumatoid arthritis, anti-MIF antibodies or genetic MIF deficiency is associated with significant inhibition of disease [40, 41]. Carriage of MIF −173 ^*^C has also been found to be associated with an increased risk of adult inflammatory arthritis [42] and juvenile idiopathic arthritis [35]. Amoli et al. have shown that MIF −173 ^*^C confers an increased risk of erythema nodosum in patients with biopsy-proven sarcoidosis [43].

Currently the mechanism of why MIF −173 ^*^C is associated with higher MIF levels in CHD patients is unclear. By using the pGL3 luciferase reporter system in A549 (an epithelial cell line) and CEM C7A cells (a T lymphoblast cell line), Donn et al. demonstrated that the MIF −173C-luc had significantly greater luciferase activity in CEM C7A cells but lower luciferase activity in A549 cells compared with the MIF −173G-luc, indicating the effect of polymorphism of MIF on the −173 position may depend on cell types, which may have different transcription factors interacting with the MIF −173 element. Nevertheless, it is encouraging to note the association of MIF −173 ^*^C with CHD, as described herein. The polymorphisms we have identified should therefore assist in the understanding of the role of MIF in the pathogenesis of CHD. Determination of MIF levels in CHD patients will be useful in distinguishing a subgroup of patients with unfavorable prognosis. Since high level of MIF is a risk factor, agents capable of decreasing endogenous level of MIF may provide opportunity for potential prevention and therapeutic intervention for CHD patients.

## Figures and Tables

**Table 1:** Frequencies of MIF − 173G/C alleles and genotypes of CHD patients and controls.

Polymorphism	Genotypes and alleles	CHD	Control	*χ* ^2^	*P*
		*n* = 70 (%)	*n* = 186 (%)		
−173G/C	CC	10 4.3	6 3.2		
	CG	14 (20)	44 23.7		
	GG	46 65.7	136 73.1	10.646	0.005
	G	106 75.7	316 84.9		
	C	34 24.3	56 15.0	1.810	0.014

**Table 2:** Frequencies of MIF − 173G/C genotypes of CHD patients and controls.

Genotypes	CHD	Control	*χ* ^2^	*P*	OR (95% CI)
CC	10	6	8.422	0.004	5.238 (1.614–17.00)
CG	14	44			
CC	10	6	10.47	0.003	4.928 (1.697–14.30)
GG	46	136			
GG	46	136	0.03	1.0	0.941 (0.473–1.872)
CG	14	44			

**Table 3:** Comparison of clinical data between control group and CHD subgroups.

	Control^*^ (*n* = 53)	SAP (*n* = 10)	UAP (*n* = 52)	*F*/*x* ^2^	*P*
Age (years)	64.02 ± 8.57	65.9 ± 10.3	66.85 ± 10.51	1.145	0.322
Gender (male/female)	27/26	7/3	32/20	1.2	0.273
Cigarette smoker (%)	30.2%	20%	40.4%	1.201	0.273
Drinking (%)	18.9%	20%	26.9%	0.964	0.326
Hypertension (%)	66.04%	60%	69.2%	0.119	0.73
HDL-C (mmol/L)	1.01 ± 0.23	0.98 ± 0.22	0.99 ± 0.24	0.082	0.921
LDL-C (mmol/L)	2.47 ± 0.74	2.4 ± 1.43	2.42 ± 0.87	0.057	0.945
TC (mmol/L)	4.33 ± 0.83	4.22 ± 1.45	4.26 ± 1.05	0.095	0.909
TG (mmol/L)	1.83 ± 1.3	1.91 ± 1.37	1.75 ± 0.95	0.19	0.827
MIF ± SD (ng/L)	48.08 ± 6.48	61.52 ± 4.3	66.79 ± 6.29	119.97	0.000
*P* value for comparison with control	Reference	0.000	0.000		
*P* value for comparison with SAP	0.000	Reference	0.016		

^*^ *Note  1*. Due to incomplete clinical data, only 53 individuals in the control group were included.
